# A new case report of severe mucopolysaccharidosis type VII: diagnosis, treatment with haematopoietic cell transplantation and prenatal diagnosis in a second pregnancy

**DOI:** 10.1186/s13052-018-0566-x

**Published:** 2018-11-16

**Authors:** Francesca Furlan, Attilio Rovelli, Miriam Rigoldi, Mirella Filocamo, Barbara Tappino, Douglas Friday, Serena Gasperini, Silvana Mariani, Claudia Izzi, Maria Pia Bondioni, Cinzia Gellera, Anna Venerando, Nicoletta Villa, Maria del Carmen Rodriguez Perez, Fabio Pavan, Andrea Biondi, Rossella Parini

**Affiliations:** 1 Pediatric Highly Intensive Care Unit, Department of Pathophysiology and Transplantation, Università degli Studi di Milano, Fondazione IRCCS Ca’ Granda Ospedale Maggiore Policlinico, Milan, Italy; 2 Clinica Pediatrica, Fondazione MBBM, Università Milano-Bicocca, Monza, Italy; 3 Medical Genetics Unit S Gerardo Hospital, ASST Monza, Monza, Italy; 4 Centro di Diagnostica Genetica e Biochimica delle Malattie Metaboliche, Istituto Giannina Gaslini, Genoa, Italy; 5 Diagenom GmbH Robert-Koch-Str. 10, D-18059 Rostock, Germany; 6 Clinica Ostetrica Fondazione MBBM Università Milano Bicocca, Monza, Italy; 7 Prenatal Diagnosis Unit, Department of Obstetrics and Gynecology, University of Brescia, Brescia, Italy; 8 Department of Medical and Surgical Specialties, Radiological Sciences and Public Health, University of Brescia, Brescia, Italy; 9 Unit of Genetics of Neurodegenerative and Metabolic Diseases,- Fondazione IRCCS Istituto Neurologico Carlo Besta, Milan, Italy; 10 U.O. di Neonatologia e Terapia Intensiva Neonatale, Ospedale dei Bambini, ASST Spedali Civili di Brescia, Brescia, Italy; 11 Fondazione MBBM, AST San Gerardo, via Pergolesi 33, 20900 Monza, Italy

**Keywords:** NIHF, Non-immune hydrops fetalis, LSDs, MPS VII, *GUSB* gene, Beta-glucuronidase, Mucopolysaccharidosis, Haematopoietic cell transplantation, HCT

## Abstract

A new patient with severe mucopolysaccharidosis (MPS) type VII is reported. Non-immune hydrops fetalis (NIHF) was diagnosed during pregnancy. At birth, he showed generalized hydrops and dysmorphic features typical of MPS. Many diagnoses were excluded before reaching the diagnosis of MPS VII at 8 months of life. During the first year of life he had frequent respiratory infections associated with restrictive and obstructive bronchopneumopathy and underwent three surgical interventions: decompression of the spinal cord at the craniocervical junction, bilateral inguinal hernia, and bilateral clubfoot. At 14 months of life he underwent successful haematopoietic cell transplantation (HCT). During the following 10 months, his bronchopneumopathy progressively worsened, needing chronic pharmacological treatment and O_2_ administration. The patient died of respiratory insufficiency during a respiratory syncytial virus infection at 25 months of age. Molecular analysis showed the homozygous variant c.1617C > T, leading to the synonymous mutation p.Ser539=. This caused aberrant splicing with partial skipping of exon 10 (r.1616_1653del38) and complete skipping of exon 9 (r.1392_1476del85; r.1616_1653del38). No transcript of normal size was evident. The parents were both confirmed to be carriers. In a subsequent pregnancy, a prenatal diagnosis showed an affected fetus. Ultrasound examination before abortion showed NIHF. The skin and placenta examination by electron microscopy showed foamy intracytoplasmic vacuoles with a weakly electron-dense substrate. MPS VII is a very rare disease but it is possible that some cases go undiagnosed for several reasons, including that MPS VII, and other lysosomal storage diseases, are not included in the work-up for NIHF in many institutions, and the presence of anasarca at birth may be confounding for the recognition of the typical facial characteristics of the disease. This is the eighth patient affected by MPS VII who has undergone HCT. It is not possible to draw conclusions about the efficacy of HCT in MPS VII. Treatment with enzyme replacement is now available and will probably be beneficial for the patients who have a milder form with no or little cognitive involvement. Increased awareness among clinicians is needed for prompt diagnosis and to offer the correct treatment as early as possible.

## Background

Mucopolysaccharidosis (MPS) type VII, or Sly syndrome (MIM 253220), is a very rare, autosomal recessive, inherited lysosomal storage disorder with an estimated overall frequency between 1/300,000 and 1/2,000,000 [1]. It is caused by deficiency of the lysosomal enzyme β-glucuronidase (EC 3.2.1.31), which leads to the storage of glycosaminoglycans (GAGs) dermatan sulfate, heparan sulfate, chondroitin sulfate in many tissues [2]. The gene encoding β-glucuronidase (*GUSB*; MIM# 611499) is 21 kb long and contains 12 exons. The presence of unprocessed multiple pseudogenes requires particular attention in diagnostic mutation analysis. To date, 64 different mutations have been reported in the *GUSB* gene at the Human Gene Mutation Database Professional (http://www.hgmd.org).

The phenotypic characteristics of MPS VII are reported to be similar to those of MPS I and MPS II, although non-immune hydrops fetalis (NIHF) is much more frequent in MPS VII [3]. As of June 2016, according to Montano et al. [1], there were 143 MPS VII patients described in the literature with a wide spectrum of severity, from milder, late-onset forms with coarse facial features, corneal clouding and frequent upper respiratory infections but mild skeletal abnormalities and normal intellectual performances, to the more severe forms characterized by hydrops fetalis, short stature and severe skeletal dysplasia, macrocephaly, ear infections, hepatosplenomegaly, hernias, and cognitive impairment.

Few single cases or small series were reported in the literature until 2016 when Montano et al. reviewed the clinical history of 56 patients collected all over the world through a survey among medical specialists who had or had previously had MPS VII patients under their charge [1]. Among these patients, 23 (41%) had NIHF; 10 of these showed a severe fetal-neonatal presentation with early death while 13 survived longer, despite NIHF. Little is known about the clinical history of the 10 patients with early death who died prenatally or shortly after birth. Of the 13 patients with history of hydrops and longer survival, five patients received haematopoietic cell transplantation (HCT) at an age between 7 months and 7 years and three of them have survived. Two other patients are reported who received HCT at the age of 11 months and 12 years and showed apparent benefit 2 years and 31 months after HCT, respectively [4, 5].

Here, we describe the clinical history of a new severe case, from consanguineous healthy parents, who was treated with HCT. The results of the prenatal diagnosis of a second pregnancy of this couple are also shown.

## Case presentation

### First year of life

The proband is a male first child of first-degree cousin parents from Pakistan (Punjabi ethnic origin). Fetal ultrasound during pregnancy revealed a bilateral clubfoot and NIHF (hydrothorax + ascites). For this reason, amniocentesis for karyotyping was performed and it gave a normal result: 46,XY; maternal-fetal infections and immune-haematological diseases were excluded.

**Fig. 1:**
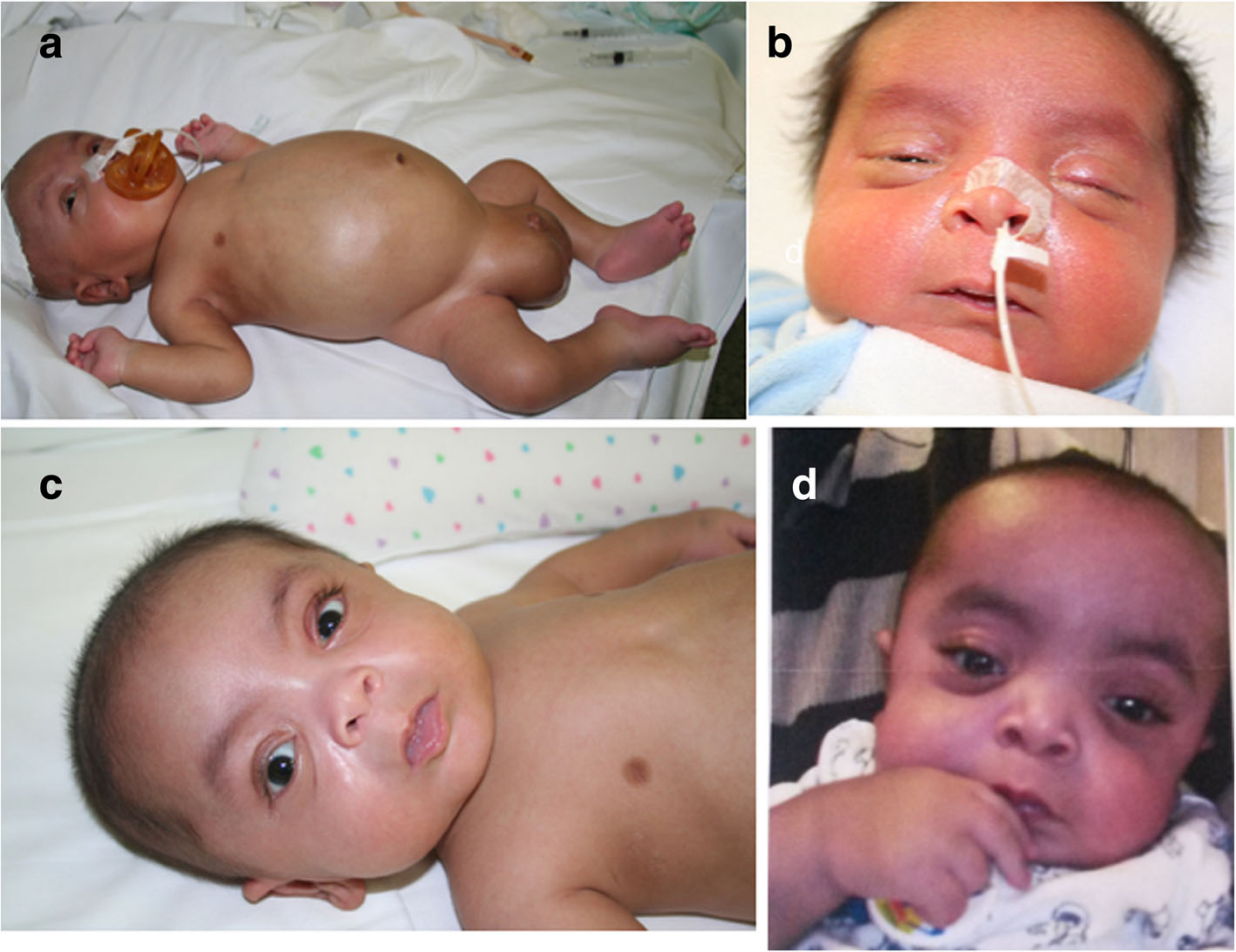
The MPS VII patient at 20 days of age (**a**, **b**), 3 months (**c**) and 8 months (**d**). Note ascites, hydrocele, and feet and face oedema (**a**, **b**), and typical dysmorphic appearance of the facies at 3 and 8 months of age

The child was born by caesarean section at 32.5 weeks. His weight was 3613 g (he had generalized oedema), length was 52 cm, and cranial circumference was 36 cm (SDs + 5.6, + 4.0, and + 4.4, respectively, according to Olsen et al. [6]). The Apgar score was 4 at the first minute and the baby was intubated. He had respiratory distress with bilateral hydrothorax that needed right-side drainage for 12 days and mechanical ventilation for 8 days, ascites, and bilateral massive hydrocele. He also exhibited brachycephaly and bilateral clubfoot. No infectious or haematological diseases were seen in the baby. Figure 1 shows the infant at 20 days of life with a mildly coarse face and generalized oedema (Fig. 1a, b).

At 1 month of age, metabolic screening on the urine showed normal free sialic acid (109 mmol/mol creatinine with normal value < 123) while the conjugated and total sialic acid were increased (811 mmol/mol creatinine with normal value < 343 and 920 mmol/mol creatinine with normal value < 454, respectively), and increased GAGs (357 mg/g creatinine with normal value 5.9–60). These results prompted the enzyme analysis on fibroblast culture for MPS I, MPS II, MPS IVA and IVB, MPS VI, sialidosis, and mucolipidosis II, which were all normal.

During the following few months, the clinical evaluation showed worsening of the facial dysmorphism (see Fig. 1c, d), persistence of hydrocele, brachycephaly and bilateral clubfoot, pectus carinatum, gibbus, hepatomegaly, bilateral inguinal hernias, and joint stiffness; a restrictive chest wall deformity was observed with very limited or absent expansion of the cage and only diaphragmatic breathing. At 3 months of age he had axial hypotonia (he did not hold his head up), mild hypertonia in the upper limbs, and moderate hypertonia in the lower limbs. At the same age, heart and kidney functions were normal. Periodic abdominal ultrasound examinations showed progressive disappearance of ascites over 6 months. Brain stem evoked potentials evidenced bilateral severe hypoacusia while the ophthalmological evaluation was normal.

**Fig. 2:**
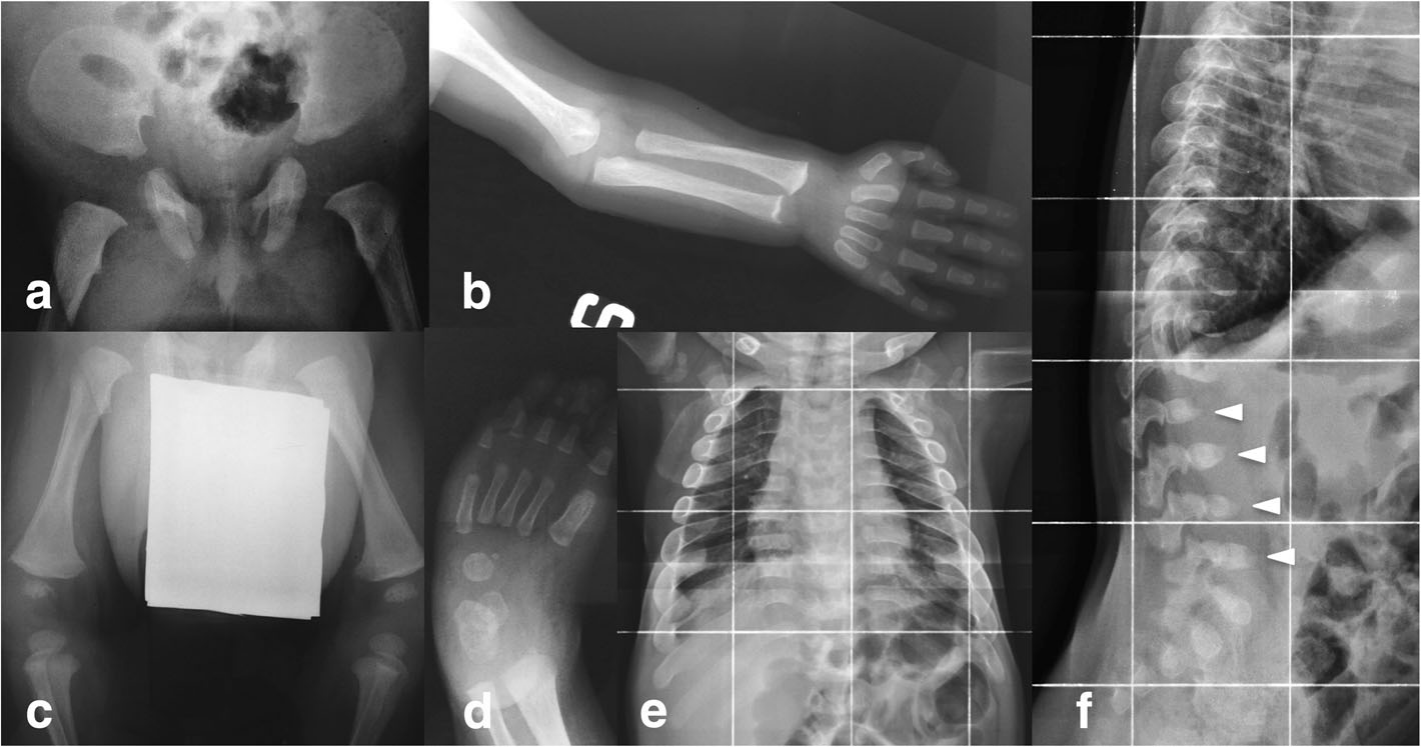
X-rays of an MPS VII infant at the age of 3 months (**a**–**d**) and 12 months (**e**, **f**) showing generalized skeletal dysplasia (dysostosis multiplex). The pelvis (**a**) shows typical imaging features characterized by rounded iliac wings and inferior tapering of the ilia with an undeveloped acetabulum; proximal epiphysis of the femurs are not ossified. The femurs (**c**) are short with hypoplastic epiphyses, similarly to the upper left limb (**b**) in which cortical thinning, flared metaphysis, and metacarpal widening are also identifiable. The hand (**b**) and foot (**d**) are typically dysmorphic: broad and short metacarpals and bullet-shaped phalanges. In the antero-posterior projection of the thorax (**e**), a marked thickening of the ribs is evident (oar shaped ribs). In the latero-lateral projection of the dorsal and lumbar spine (**f**), the lumbar vertebral bodies from L2 to L5 are markedly wedge deformed with anterior beaking aspect (arrows), and with angulation of the dorsal-lumbar tract

**Fig. 3:**
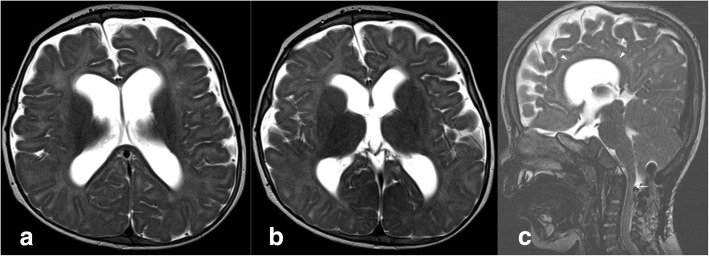
Brain MRI of an MPS VII patient at 12 months acquired post-surgery. On MR T2-weighted images acquired on the axial plane (**a**, **b**) through lateral ventricles and basal ganglia, enlargement of subarachnoid spaces and dilatation of the ventricular system are visible. On the sagittal plane (**c**) the corpus callosum is thinned and dysmorphic (arrowheads), and at the C1–C2 level, a cervical canal stenosis is still detectable (arrow)

**Fig. 4:**
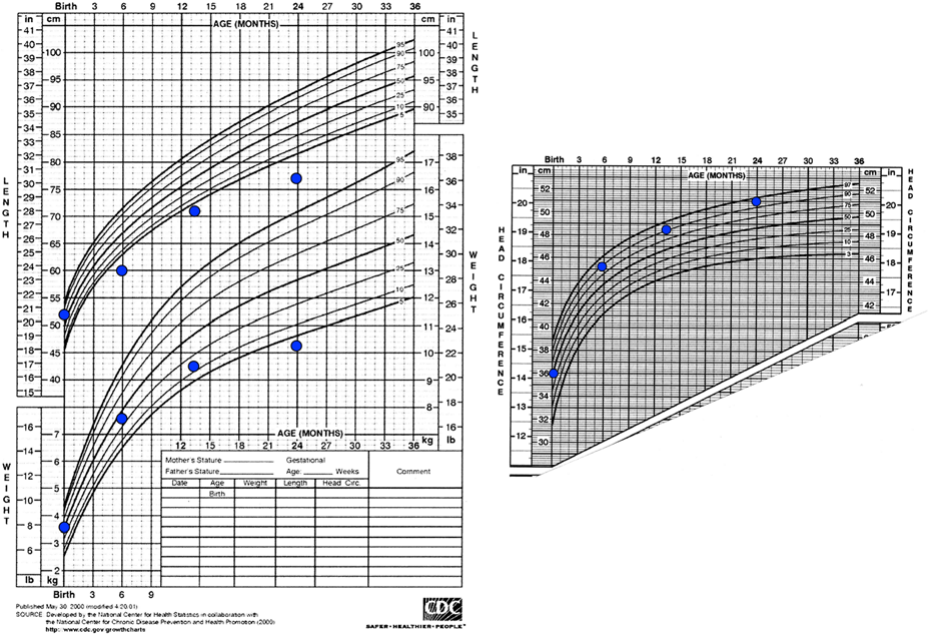
Growth centiles of the MPS VII patient showing a progressive decrease in height and weight and mild increase in head circumference

The skeleton x-ray showed dysostosis multiplex. In particular, spine abnormalities (craniocervical junction malformation, wedge-shaped L3 and L4, kyphosis, and scoliosis), oar ribs, hypoplasia of the inferior portion of the iliac bones and flared iliac wings, and squat femurs were seen (Fig. 2); brain magnetic resonance imaging (MRI) showed enlargement of the subarachnoid spaces and ventriculomegaly with spinal canal stenosis at the C1 level (Fig. 3). Growth curves of the patient are presented in (Fig. 4).

All these findings, together with his clinical history, supported the suspicion of MPS, and MPS VII was eventually investigated. The enzymatic assay for β-glucuronidase, performed in cultured fibroblasts, revealed a β-glucuronidase activity of 22 nmol/h/mg (normal value 200–600; residual activity 5.5%) and confirmed the diagnosis of MPS VII.

During the first year of life, the patient had frequent respiratory infections associated with wheezing and desaturation and underwent three surgical interventions: at 5 months for decompression of the spinal cord at the craniocervical junction, and at 10 and 12 months, respectively, for bilateral inguinal hernia and bilateral clubfoot.

At 12 months, a Griffiths test showed mildly delayed psychomotor development (general quotient 70).

### Haematopoietic cell transplantation (HCT) and second year of life

At 14 months, the patient underwent successful HCT from an unrelated 5/6 human leukocyte antigen (HLA)-matched cord blood unit (total nucleated cells 7.3 × 10^7^/kg, CD34^+^ cells 1.83 × 10^5^/kg). The conditioning regimen included Busulfan and cyclophosphamide and graft-versus-host disease (GvHD) prophylaxis, anti-thymocyte globulin (ATG), cyclosporine, and methylprednisolone. Engraftment was achieved on day 31. The post-transplant course was complicated by rotavirus gut infection, *Staphylococcus aureus* bacteraemia, cytomegalovirus reactivation (the recipient was positive), and acute grade III GvHD, which all resolved. Chimerism had been continuously documented as 100% donor. He made developmental improvements and started walking independently at 20 months of life.

In the second year of life, he developed chronic pulmonary insufficiency with polypnoea and wheezing, needing chronic therapy with inhaled salbutamol and a positive end-expiratory pressure (PEEP) mask. Frequent acute exacerbations with deep desaturations were also observed with fever and/or infections and treated with an increased dosage of beta2 agonist plus anticholinergic bronchodilator, corticosteroids, and O_2_ therapy. Airway computed tomography (CT) scans at 13 and 20 months of life were similar and showed a restricted rib cage with multiple dystelectatic areas of the lungs and no tracheal abnormalities. From 20 months of age (6 months after transplantation) his maximum O_2_ saturation outside infection was 93–94% with lower values during sleep; from then on, he started chronic O_2_ administration at home during the night. At month 9 after HCT he started chronic betamethasone and O_2_ administration the whole day due to worsening respiratory distress. At 11 months after transplantation, the child had a new acute episode of respiratory distress that required hospitalisation in the Paediatric Intensive Care Unit with intubation and mechanical ventilation. Infection with respiratory syncytial virus was detected and, unfortunately, his respiratory insufficiency did not improve and he deceased at 25 months of age.

### Molecular studies

DNA analysis of the proband identified the homozygous genetic variant c.1617C > T, leading to the synonymous mutation p.Ser539=. This nucleotide variation, generating a 5′ splice site (GT) in a non-canonical exonic position, had previously been reported as causing an aberrant partial skipping of the exon 10 (r.1616_1653del38) in a compound heterozygous patient [7]. To confirm the aberrant splicing, reverse transcriptase polymerase chain reaction (RT-PCR) was conducted on *GUSB* mRNA extracted from the proband’s fibroblast culture. The RT-PCR and sequence analyses showed the expected partial skipping of exon 10 (r.1616_1653del38) already reported by Yamada et al. [7] and, in addition, an abnormal shorter product in which complete skipping of exon 9 also occurred (r.1392_1476del85;r.1616_1653del38). No transcript of normal size was evident. The parents were both carriers of the c.1617C > T mutation.

### Genetic counselling and pre-natal diagnosis

**Fig. 5:**
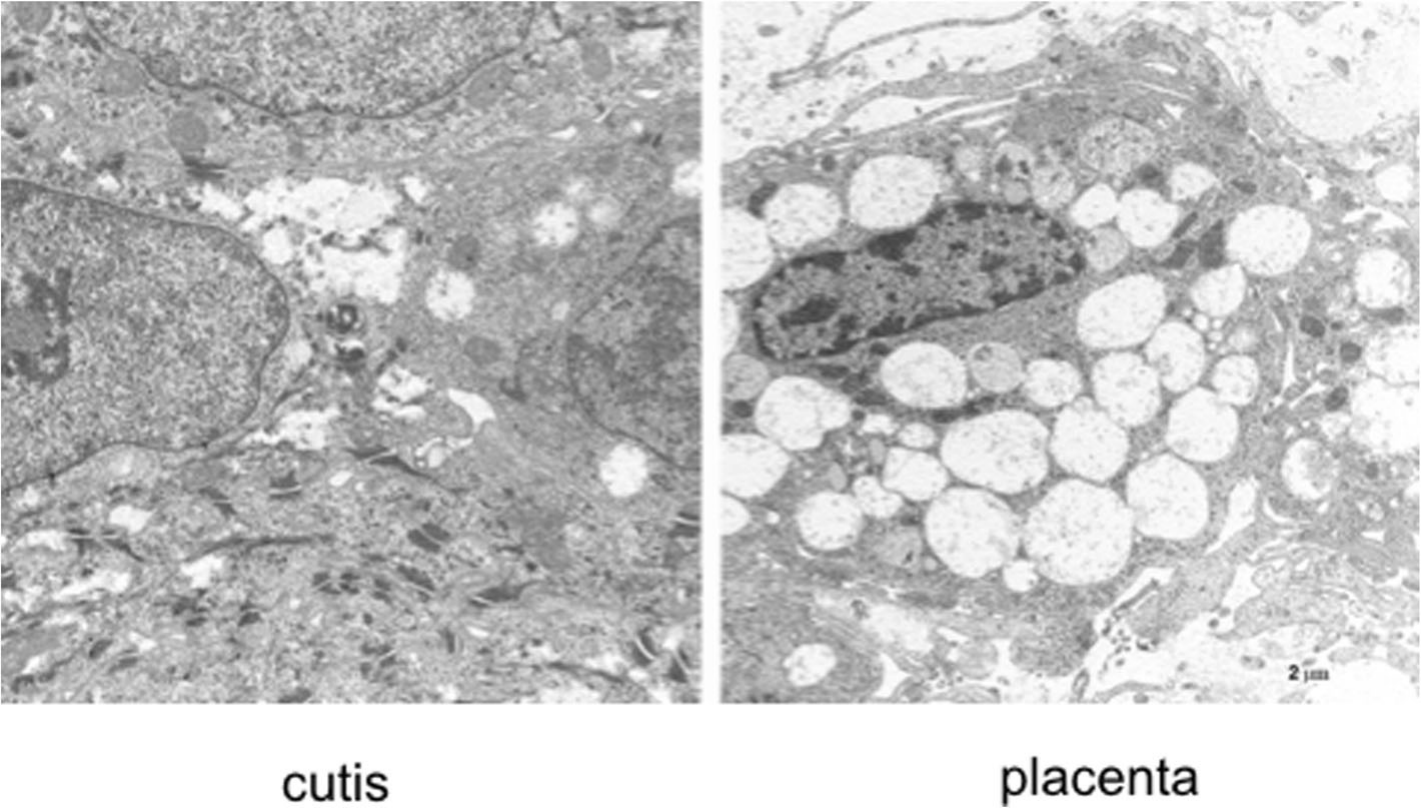
Electron microscopy of the skin (left) and placenta (right) of an 18-week MPS VII fetus, showing the presence of many foamy intracytoplasmatic vacuoles with a weakly electron-dense substrate

Based on the information provided during the genetic counselling, the couple requested pre-natal diagnosis in a subsequent pregnancy. Chorionic villus sampling (CVS) was performed. The molecular analysis revealed that the DNA from the CVS carried the homozygous c.1617C > T variant, as the affected proband. The couple decided to abort. At 18 weeks of gestation, ultrasound examination before abortion showed fetal hydrops, skin oedema, bilateral pleural effusion, and thickness of the placenta. No structural abnormalities of the fetal organs were observed and a normal volume of amniotic fluid was present. The skin and placenta examination by electron microscopy showed the presence of foamy cytoplasmic vacuoles with a weakly electron-dense substrate (Fig. 5), in accordance with the literature [7, 8].

## Discussion and conclusions

To our knowledge, this is the first report of an MPS VII patient ever diagnosed in Italy. However, the ethnic background of the patient was not European but Southern Asian. Until now, only a subject with a pseudodeficiency of the gene (Asp152Asn) is described with Italian origin in the literature [9]. This variant, Asp152Asn, shows a European allele frequency of 0.00172 versus that found in the Asian (0.00006224), Latino (0.0003956), and African (0.000206) populations, respectively (data from ExAC Browser Beta http://exac.broadinstitute.org/). We suppose that MPS VII is very rare in Italy, as in other countries in western Europe, but attenuated cases might have been overlooked. MPS VII is an ultra-rare disease and is probably less known and diagnosed than the other MPS types. [1]. Our patient was tested for many other metabolic disorders before being tested for MPS VII. It is possible that the presence of anasarca in many MPS VII newborns may prevent recognition based on the coarse facies and contributes to delayed diagnosis. As recently reported in a systematic review aiming to evaluate the incidence of lysosomal storage disorders (LSDs) in 54 case series of NIHF published from 1979 through January 2014 [10], LSDs were not tested in 72% of the papers reporting work-up for NIHF. Nonetheless, there are at least fourteen LSDs that are a possible cause of NIHF [11] and among them MPS VII is the most frequent accounting alone for 20% [10]. It is therefore evident that MPS VII should be tested in all cases of NIHF, even though it is an ultra-rare disease. The availability of new techniques such as next-generation sequencing (NGS) might help to improve the number of diagnoses in this field; a specific NGS panel for NIHF, testing not only LSDs but also the other metabolic disturbances known to be a cause of NIHF, such as CDG syndromes, peroxisomal disorders, disturbances of cholesterol metabolism, and others, could be used as a first-line investigation technique as recently proposed by Sudrié-Arnaud et al. [12].

According to the classification of Montano et al. [1], the proband had the severe infantile form. He was able to recover from NIHF and also survived the three surgical interventions performed in his first year of life. His pulmonary involvement was severe and it was the cause of his death. Reasonably, rather than a consequence of his HCT, his bronchopneumopathy can be interpreted as an expression of a chronic bronchopulmonary disease, favoured by prematurity, NIHF with pleural effusion, mechanical ventilation, and chest wall deformities which prevented a normal chest expansion. Interestingly, 71% of the patients in the study of Montano et al. [1] had decreased pulmonary function (both restrictive and obstructive); they had thoracic deformities contributing to restrictive airway disease, leading to chronic hypoventilation with low forced vital capacity, but also individual patients were reported with severe pulmonary disease (bronchopulmonary dysplasia with fibrosis), recurrent pneumothoraces, interstitial lung disease, and prolonged oxygen dependency [1]. It appears that severe bronchopulmonary involvement in MPS VII is similar to that found in MPS II [13] but is probably more frequent than in MPS I [14].

Seven MPS VII patients are described so far who have undergone HCT (Table 1) [1, 4, 5]. Of these, two were transplanted at a very late age (7 and 12 years). The age of transplantation is not known for a third patient. The outcome is available for three of the other four patients, although only the short-term outcome for two of them. Our patient is the eighth who was transplanted; he underwent the procedure at a reasonable age and did not have any initial severe adverse events. His clinical picture was, however, dominated by the respiratory insufficiency, which was probably multifactorial, and his outcome was unfavourable. The data available are too scarce, and the patients transplanted too few, to allow a conclusion about the efficacy of HCT in MPS VII. Enzyme replacement therapy (ERT), which has recently been approved by the Food and Drug Administration (FDA) in November 2017 for paediatric and adult patients with MPS VII [15], seemed beneficial on the severe bronchopulmonary involvement as reported for the first patient who underwent ERT [16]; in the blind-start study recently performed on 12 subjects by Harmatz et al. [17], most patients could not be tested for pulmonary function and, of the 2 who were tested, one worsened and the other did not improve.

**Table 1:** Mucopolysaccharidosis VII patients reported in the literature who underwent HCT

Patient	Gender	Age at onset	Age at diagnosis	Hydrops	Age at HCT	Post-HCT outcome	Reference
1	F	Birth	1 year 10 months	No	2 years (failed) and 4 years	Alive at 1 month	[1]
2	M	Unknown	2 years	No	After 7 years	Died of complications from HCT	[1]
3	M	4 months	4 months^a^	No	Unknown	Died a few years after HCT (no follow-up data)	[1]
4	M	Prenatal	26 months	Yes	3 years	Moderate clinical phenotype at 15 years	[1]
5	F	Prenatal	2 weeks	Yes	7 months	Normal developmental milestones reached at 15 months	[1]
6	F	Birth	Unknown	No	11 months	Well 2 years after HCT	[4]
7	F	Birth	1 month	No	12 years	Improvement of motor functions after 18 months. No more ENT infections	[5]
8	M	Prenatal	8 months	Yes	14 months	Died at 25 months of respiratory failure	Present study

*ENT* ear, nose, and throat, *F* female, *HCT* haematopoietic cell transplantation, *M* male

^a^Previously affected sibling

The major limitation of ERT for MPS is that it does not cross the blood–brain barrier preventing any effect on the central nervous system (CNS) in severely affected patients who show cognitive involvement (see Concolino et al. in this Supplement for details [18]).

The synonymous mutation (c.1617C > T; p.Ser539=), previously reported in a compound heterozygous patient [7], is first described in the homozygous state in the present report.

In addition, the RT-PCR analysis on the proband’s samples provided new insights on the effect of the substitution c.1617C > T, creating a non-canonical intra-exonic 5′ splice site (GT) on *GUSB* mRNA processing. Indeed, the results revealed not only the presence of the previously described shorter transcript with partial skipping of exon 10 [7], but also an additional shorter GUSB transcript in which complete skipping of exon 9 occurred upstream of the partial skipping of exon 10.

The ultrastructural appearance of the fetal tissues (Fig. 5) with numerous cytoplasmic vacuoles was consistent with previous findings in the literature [7, 8].

Over the last few years, the use of multiple diagnostic NGS-based panels has become available for genetic disorders. Therefore, in the near future, it is possible that NGS techniques could help clinicians in performing earlier diagnoses of ultra-rare diseases, a fundamental condition for timely access of patients with a progressive disease to therapies that offer hope for a better prognosis.

## Abbreviations

CVS: Chorionic villus sampling
ERT: Enzyme replacement therapy
GAG: Glycosaminoglycan
GvHD: Graft-versus-host disease
HCT: Haematopoietic cell transplantation
LSD: Lysosomal storage disorder
MPS: Mucopolysaccharidosi(e)s
NGS: Next-generation sequencing
NIHF: Non-immune hydrops fetalis
RT-PCR: Reverse transcriptase polymerase chain reaction
